# The current role and future prospectives of functional parameters by diffusion weighted imaging in the assessment of histologic grade of HCC

**DOI:** 10.1186/s13027-018-0194-5

**Published:** 2018-07-03

**Authors:** Vincenza Granata, Roberta Fusco, Salvatore Filice, Orlando Catalano, Mauro Piccirillo, Raffaele Palaia, Francesco Izzo, Antonella Petrillo

**Affiliations:** 1 Radiology Division, Istituto Nazionale Tumori IRCCS Fondazione G. Pascale – IRCCS di Napoli, via Mariano Semmola, I-80131 Naples, Italy; 2 Hepatobiliary Surgical Oncology Division, Istituto Nazionale Tumori IRCCS Fondazione G. Pascale – IRCCS di Napoli, via Mariano Semmola, I-80131 Naples, Italy

**Keywords:** HCC, Magnetic resonance imaging, Diffusion weighted imaging, Histologic grade

## Abstract

Hepatocellular carcinoma (HCC) is one of the most common human solid malignancies worldwide. Although the MRI is the technique that is best adapted to characterize HCC, there is not an agreement regarding the study protocol and even what the role of Diffusion-weighted imaging (DWI). The possibility that imaging study can correlate to histologic grade to selecting the therapeutic strategy would be valuable in helping to direct the proper management of HCC. Apparent Diffusion Coefficient (ADC) and IVIM-derived perfusion fraction (fp) and tissue diffusivity (Dt) values of HCC showed significantly better diagnostic performance in differentiating high-grade HCC from low-grade HCC, and significant correlation was observed between ADC, fp, Dt and histological grade.

## Background

Hepatocellular carcinoma (HCC) is the most common primitive hepatic cancer [1, 2]. Imaging surveillance is a widely established tool that increases the probability of early detection of HCC, which is mandatory on patient at risk for this tumor since the treatment of HCC is different to other hepatic lesions [1]. According to the guidelines of National Comprehensive Cancer Network (NCCN) [3] and of European Association for the Study of the Liver (EASL) and American Association for the Study Liver Diseases National Comprehensive Cancer Network (AASLD), during the phase of HCC characterization, the diagnostic criteria should be used only for cirrhotic patients [4]. However, the up-to-date imaging-based criteria have several limits, counting the absence of recognized agreement concerning the precise descriptions of imaging features, binary classification (either definite or not definite HCC), and disappointment to report non-HCC malignancies and vascular involvement [5]. Therefore, the American College of Radiology (ACR) has encouraged the use of Liver Imaging Reporting and Data System (LI-RADS) for the reading, recording and data collection of HCC nodules [6, 7]. Although imaging techniques allow identifying and characterizing of liver nodules with a higher diagnostic accuracy and Magnetic Resonance Imaging (MRI) is the diagnostic tool that should be chosen to survive HCC patients [8–10], however the gold standard to characterize liver lesions is still biopsy [11]. In fact, until now, histological analysis is the unique technique that allow to identify the histologic grade of HCC, that is one of the most predictive factors of survival for HCC patients [11]. During the last years, the possibility to obtain functional data by Diffusion-weighted imaging (DWI), it has seen born a great interest on this technique. DWI has been applied to liver imaging as an excellent tool for detection and characterization of focal liver lesions, increasing clinical confidence and decreasing false positives [11–14]. Oncology is a major field of application of DWI. The analysis of DW images can be done qualitatively and quantitatively, through the apparent diffusion coefficient (ADC) map. Eco Planar Imaging (EPI) sequences are widely used for DWI, which are basically T2-W sequences, acquired with single shot technique and FS. Different series of DW images are acquired through modification of the gradient strength and magnitude, referred as b-value. One series should be obtained with a b-value of 0, meaning no gradient is applied and consequently no diffusion information is retrieved, giving similar information as T2 FS sequences. Another series should be obtained with a low b-value (b < 100), for lesion detection, while series obtained with a high b-value (such as b = 800) are important for liver lesion characterization [11–14]. DWI signal depends on the water mobility that is related to tissue characteristics [11]. Diffusion is quantified by a diffusion coefficient, the ADC. The ADC map is the graphical representation of the ratio of DW signal intensities and its measurements may discriminate between benign and malignant lesions. The ADC measurements are correlated to the sequence acquisition protocol and suffer from a lack of reproducibility, especially in respiratory triggering techniques, nodules of left liver lobe, smaller size and lesion heterogeneity [11]. Accurate estimation of ADC can be improved by acquiring a large number of b-values. ADC low values mean restricted diffusion, high ADC values mean free or unimpeded diffusion. Malignant tissue shows signal hyperintensity in DWI and signal hypointensity in the apparent diffusion coefficient (ADC) map [11]. Several researchers investigated, therefore, the values of ADC for lesion characterization. The ADC values for malignant lesions vary in literature widely and show a significant overlap with benign and other malignant lesions [11–14]. Le Bihan et al. as a first assessed the intravoxel incoherent motion (IVIM) and evaluated a more sophisticated method to define the relationship between signal attenuation and increasing b value that separately reproduce tissue diffusivity and tissue perfusion [12, 13]. IVIM data can be assessed qualitatively and quantitatively. The lesion characterization is done easier by quantitative data, while qualitatively method helped the detection of nodule [13]. In clinical practice DWI is widely employed after neoadjuvant therapy [14] or ablative techniques [15], to assess the efficacy of treatment. An emerging field of application of DWI is the evaluation of histological grade of the tumor [16]. Several researches have assessed the relationship between functional parameters obtained by DWI and histological grade of HCC [16–19]. Considering that the histologic grade of HCC is one of the most predictive factors of reappearance and survival after treatment and transplantation [16–19], the probability that imaging analysis could be associated to the histologic grade to selecting the therapeutic approach should guide the proper treatment of patient.

Our purpose is reporting an overview and update of the role of DWI in assessment of histologic grade of HCC.

## Methods

This overview and update is the result of autonomous studies without protocol and registration number.

### Search criterion

We evaluated several electronic databases, PubMed (US National Library of Medicine, http://www.ncbi.nlm.nih.gov/pubmed), Scopus (Elsevier, http://www.scopus.com/), Web of Science (Thomson Reuters, http://apps.webofknowledge.com/) and Google Scholar (https://scholar.google.it/), using as search criteria the following key words: “hepatocellular carcinoma” AND “diffusion magnetic resonance imaging” AND “histologic grade”, “hepatocellular carcinoma” AND “intravoxel incoherent motion” AND “histologic grade”, “hepatocellular carcinoma” AND “multimodal imaging” AND “histologic grade”. Our analysis enclosed the time between January 2000 and October 2017. Also, we evaluated the references of the searched studies for documents not indexed in the electronic databases. We retained solely the papers recording DWI results in the evaluation of histologic grade of HCC. Articles published in the English language from January 2000 to October 2017 were included. The absence of full text, overview analysis and conference papers were considered as exclusion criteria.

### Histological grading assessment in HCC

The classical and most commonly adopted grading system for HCC is Edmondson–Steiner (ES) [20], published in the 1954 that organized the tumors in 4-tier histological grade distribution (Table 1). In contrast, and most likely due to differences from the ES classification, the World Health Organization (WHO) classification organized tumors in 3-tiers (Table 1) [21]. Usually the researches, when WHO classification is adopted, tend to assess each grade individually (G1 × G2 × G3), while when is adopted ES classification, they dichotomize them in low (G1 + G2) and high grades (G3 + G4).

**Table 1:** Histological features according to Edmondson and Steiner (ES) and WHO classification

Classification	Grades	Architecture	Cytology	Other features
Edmondson and Steiner	I	–	–	Areas of carcinoma where distinction from hyperplastic liver is difficult
	II	Trabecular, frequent acini (lumen varying from tiny canaliculi to large thyroid-like spaces)	Resemblance to normal hepatic cells; larger nuclei; abundant acidophilic cytoplasm	Cell borders sharp and clear cut; acini containing bile or protein precipitate
	III	Distortion of trabecular structure, acini less frequent than grade II	Larger, more hyperchromatic nuclei, granular but less acidophilic cytoplasm	Acini are less frequent; tumor giant cells may be numerous
	IV	Medullary, less trabeculae, rare acini	Highly hyperchromatic nuclei, scanty cytoplasm, with fewer granules	Loss of cell cohesiveness; giant, spindle or short-plump cells can be found
World Health Organization	Well differentiated	Thin trabecular, frequent acinar structures	Minimal atypia	Fatty change is frequent
	Moderately differentiated	Trabecular (3 or more cells in thickness) and acinar	Abundant eosinophilic cytoplasm, round nuclei with distinct nucleoli	Bile or proteinaceous fluid within acini
	Poorly differentiated	Solid	Moderate to marked pleomorphism	Absence of sinusoid-like blood spaces
	Undifferentiated	Solid	Little cytoplasm, spindle, or round-shaped cells	–

## Results

**Fig. 1:**
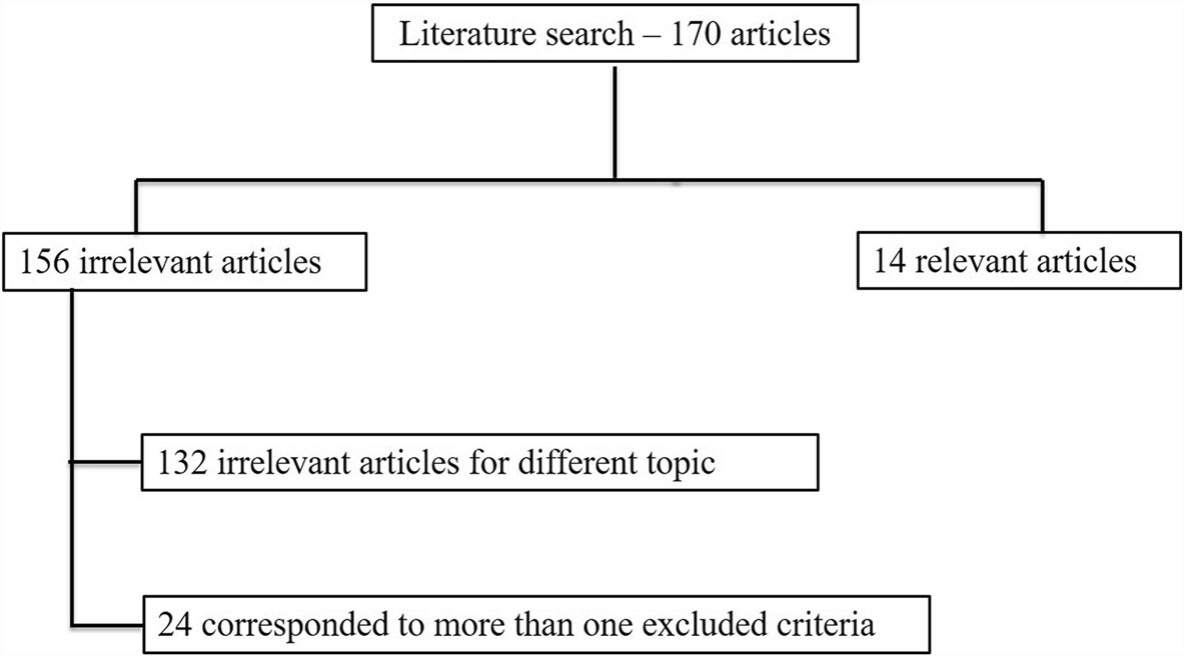
Included and excluded studies in systematic review

We collected 170 studies from the literature research from January 2000 to October 2017 considering the key words described above. However, 132 papers have different topic respect to correlation between HCC histologic grade and DWI and 24 studies corresponded to more than one excluded criteria. Therefore, fourteen articles were included at the end (Fig. 1).

## Discussion

**Fig. 2:**
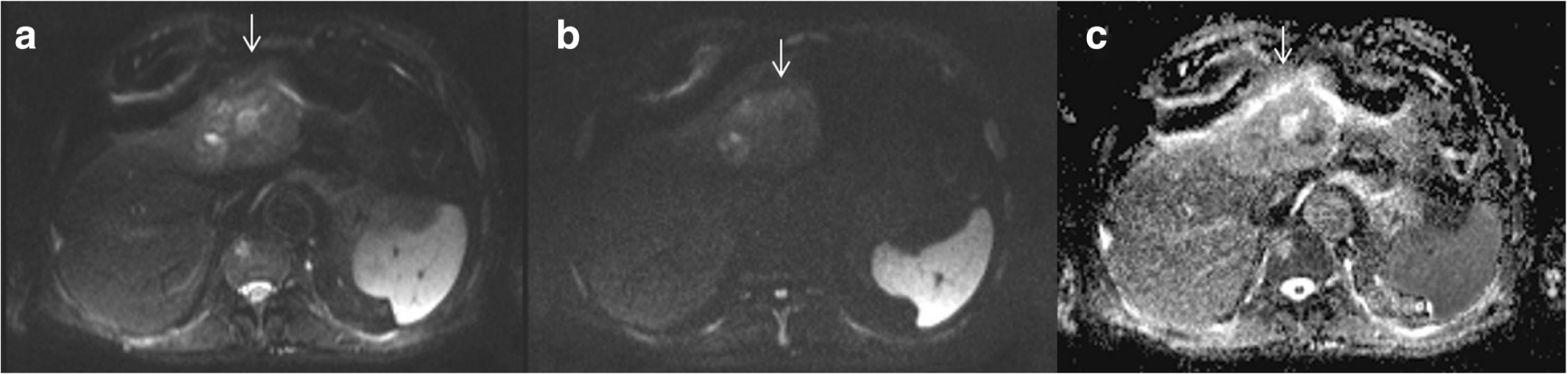
High-grade HCC. DWI sequences: in A b50 s/mm^2^; in B b800 s/mm2 and in C ADC map

**Fig. 3:**
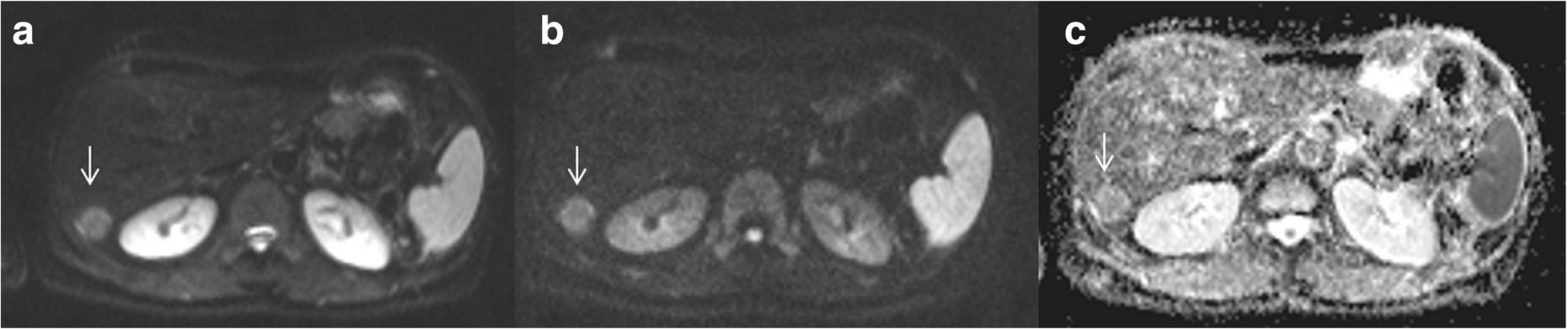
Low-grade HCC. DWI sequences: in A b50 s/mm^2^; in B b800 s/mm2 and in C ADC map

The accurate detection of histologic grade of HCC is thought a main parameter in planning of the therapeutic approach [22]. Seeing that histological analysis of small doubtful nodule is often not feasible due to their location, the role of pre-operative imaging for the assessment of well, moderate and poorly differentiated HCCs is crucial [23]. DWI is a functional MRI technique that allows quantitative evaluation of water proton diffusion in tissues. HCC is characterized by increased cellularity and, thus, have restricted diffusion [11]. Intravoxel incoherent motion (IVIM) is a recently developed DWI-derived tool. IVIM can separate the effects of perfusion-related diffusion from pure molecular diffusion [11]. DWI and IVIM enable improved detection and characterization of HCC [11]. According to Granata et al. [11] DWI and IVIM should be a role in predicting of the histological grade of HCC. In fact, they showed a good correlation between ADC, fp (perfusion fraction), and Dt (tissue diffusivity) and tumoral grading. ROC analyses showed that an ADC value of 2.11 × 10–3 mm2/sec, an fp value of 47,33% and an Dt value of 0.94 × 10–3 mm2/sec were the most accurate cut off levels to discriminate high grade versus low grade, with a sensitivity and specificity for ADC of 100 and 100%, for fp of 100 and 89%, for Dt of 100 and 74%, respectively. Guo et al. assessed the relationships of signal intensity (SI) and ADC with the histological grade in 27 resected HCC patients. They showed that there were no significant differences in ADC parameters or SI between higher or lower grade of HCC nodules. In fact the overall ADC rate for all cases was 1.28 ± 0.19 × 10–3 mm2/s. The ADC was 1.16 ± 0.16 × 10–3 mm2/s for poorly differentiated nodules, lower than the well [1.43 ± 0.09 × 10–3 mm2/s] and moderately [1.34 ± 0.19 × 10–3 mm2/s] differentiated HCCs. The overall SI value was 75.66 ± 32.94. The mean SI value for the moderately differentiated HCCs was 54.37 ± 28.37, lower than the well (90.78 ± 27.49) and poorly (86.77 ± 31.51) differentiated [16]. Nakanishi et al. showed not only the utility of DWI for histological tumor grading, but also that ADC should be used as a preoperative prediction of early recurrence [22]. DWI is a valuable diagnostic tool, that allows not invasively characterizing biological tissues by measurement of properties of water diffusion, however there are results which are in contrast each one [12–19, 24–30]. Chen et al., in a meta-analysis, found that for differentiating well differentiated lesions from higher grades, DWI showed a low sensitivity (54%), high specificity (90%), and an excellent diagnostic performance (area under curve (AUC) = 0.9311). Conversely, in differentiating poorly differentiated lesion from lower grades, the sensitivity was 84%, the specificity 48%, showing a moderately high diagnostic performance [18]. Nasu et al. evaluated 125 resected HCCs showing no association between histological grade and ADC, while they found that SI of the HCC increased in higher grade [23]. Instead, Muhi et al. found significant changes in SI and ADC between different grades of 98 HCC nodules, although there was still considerable overlapping [24]. Nishie et al. found a relationship between ADC parameters and HCC histological data, but the difference was significant only between well-differentiated and poorly differentiated lesions [25]. Recent technique advance has endorsed the application of IVIM in predicting the histological grade of HCC [17]. By using the IVIM model diffusion features can be disconnected from pseudo diffusion caused by perfusion [12, 13]. According to Woo et al. IVIM-derived diffusion values (diffusion coefficient, Dt) had considerably higher diagnostic performance compared to ADC in discerning high grade (Fig. 2) from low grade HCC (Fig. 3) [17]. Conversely Granata et al. showed that the ADC had the best diagnostic performance, in comparison of fp and Dt [11]. However the mayor limit of DWI and IVIM parameters to discriminate the histological grade of HCC, as suggested by Ichikawa et al., is depending on the fitting methods used to obtained functional parameters, thus the fitting would be robust even though some errors might have occurred during image acquisition [29]. A prospective study with a larger cohort would be necessary to confirm the usefulness of the IVIM parameters for distinguishing poorly differentiated HCCs from other HCC grades and to establish the advantages of this method [29].

Several tumor features evaluated by imaging techniques can be associated with HCC prognosis after treatment [31]. Microvascular invasion (MVI), defined as microscopically detected tumor thrombi within small tumor or peritumoral vessels, to day, is considered a major risk factor of recurrence [31]. DWI and DWI-based approaches (IVIM and Kurtosis) play a pivotal role in assessment of MVI. Several researches have shown that higher tumor-to-liver signal intensity ratio and lower ADCs value can predict MVI [32–34]. This could be due to higher cellularity with restricted diffusion and decreased perfusion in MVI HCCs compared with no MVI HCC. Wang et al. assessed the role of kurtosis in HCC showing that mean kurtosis values increased in MVI positive patients so that those can be independent risk factors for MVI [35].

Another field of attention is the role of DWI in the assessment of immunotherapy. To date at the best of our knowledge there are not present in literature studies that describe the role of DWI in the assessment of HCC response after immunotherapy. However, Qin et al. [36] report the promising results of DWI in glioblastoma patients subjected to anti-PD1 therapy in order to differentiate patients who derive therapeutic benefit from those who do not. Preliminary data from advanced MRI assessment suggests that increase in volume of abnormal tissue with contrast enhancement, edema, and intermediate ADC occurs in most patients during the initial months of anti-PD1 ± anti-CTLA-4 immunotherapy. Among patients who appear to achieve therapeutic benefit, subsequent improvement in these MRI markers was observed. Their findings suggest that volumetric change in ADC may correlate better with therapeutic benefit than RANO criteria measures. Therefore, future endpoint could be the evaluation of HCC tissue and in particular of immune cell infiltrate using DWI after immunotherapy [36].

Although there are the great advantages due to DWI and DWI-based approaches in detection and characterization of HCC, and DWI has been included in the Liver Imaging Reporting and Data System [9, 10], these approaches have several limitations. First, the performance of DWI for detection could be degraded due to the not standardized acquisition protocol, including the determination of optimal b values and breathing techniques across different modalities and medical centers. Therefore, universal thresholds for ADC and other quantitative parameters may not be acquirable. Second, DWI is sensitive to motion artifact; thus, detection and characterization of lesions can be mostly affected in the presence of motion artifacts [29].

## Conclusion

The histologic grade of HCC is one of the most prognostic features of reappearance and survival after surgical treatment and transplantation. The probability that the imaging could identify the histologic grade of HCC should be a useful tool to guide the patient management. In this context, MRI study with the DWI sequences should be the method to choose because the ADC and IVIM-derived parameters are related to the histological grade of HCC. However, a larger study group would be necessary to confirm the usefulness of the IVIM parameters for distinguishing poorly differentiated HCCs from other HCC grades.

## Abbreviations

AASLD: Association for the Study Liver Diseases National Comprehensive Cancer Network
ACR: American College of Radiology
ADC: Apparent diffusion Coefficient
AUC: Area under curve
Dt: Tissue diffusivity
DWI: Diffusion-weighted imaging
EASL: European Association for the Study of the Liver
fp: Perfusion fraction
HCC: Hepatocellular carcinoma
IVIM: Intravoxel incoherent motion
LI-RADS: Liver Imaging Reporting and Data System
MRI: Magnetic Resonance Imaging
NCCN: National Comprehensive Cancer Network
